# Role of reactive nitrogen species in male infertility

**DOI:** 10.1186/1477-7827-10-109

**Published:** 2012-12-15

**Authors:** Sejal B Doshi, Karishma Khullar, Rakesh K Sharma, Ashok Agarwal

**Affiliations:** 1 Center for Reproductive Medicine, Cleveland Clinic, Euclid Avenue, Cleveland, 9500, OH, USA

**Keywords:** Infertility, Nitric oxide, Reactive nitrogen species, Oxidative stress

## Abstract

Reactive nitrogen species (RNS) is a subset of free oxygen radicals called reactive oxygen species (ROS). Physiological levels of ROS are necessary to maintain the reproductive functions such as cell signaling, tight junction regulation, production of hormones, capacitation, acrosomal reaction, sperm motility, and zona pellucida binding. However, an excess of RNS can adversely affect reproductive potential by causing testicular dysfunction, decreased gonadotropin secretion, and abnormal semen parameters. Because such levels of RNS have been demonstrated in males with fertility problems and routine semen analysis has not been able to accurately predict IVF outcomes, it is imperative that novel strategies be developed in order to both assess and treat oxidative stress. This article describes both physiological and pathological roles of this unique subset of ROS.

## Background

Reactive Oxygen Species (ROS) comprise a class of radical and nonradical oxygen derivatives that play a significant role in reproductive biology. Because they have an unpaired electron in their outer orbit, ROS are highly reactive and interact with a variety of lipids, proteins, and nucleic acids in the body [1-12]. Such reactions are not only harmful for reproductive potential, but they also generate more free radicals, thereby perpetuating a chain of reactions and creating high amounts of oxidative stress.

Oxidative stress (OS) results from an imbalance of free radicals and antioxidant defense mechanisms in the body. Normally, antioxidants, both enzymatic and non enzymatic, scavenge free radical species and protect the body from over-exposure to oxidative stress. For instance, the antioxidant Vitamin E has been shown to enhance the fusion of spermatozoa with the oocyte, thereby improving zona pellucida binding. Another antioxidant that has been shown to neutralize radical-induced damage to the sperm plasma membrane is albumin. However, in certain instances, the lack of antioxidants can result in oxidative stress [11].

OS has been associated with many deleterious effects on the reproductive system as well as numerous diseases, such as cancer, diabetes, varicocele, rheumatoid arthritis, AIDS, inflammation, and liver damage [4-6,10-19]. ROS, not only includes oxygen radicals such as the hydroxyl radical, superoxide radical, and hydrogen peroxide but also a subclass of nitrogen-containing compounds collectively known as reactive nitrogen species (RNS). Examples of RNS include peroxynitrite anion, nitroxyl ion, nitrosyl-containing compounds, and nitric oxide. While important for various physiological functions, RNS in excessive amounts, which contributes to nitrosative stress, may exert pathological effects on the male reproductive system [10-12,17,18,20]. In particular, RNS has recently been implicated in inducing poor sperm function and sperm fertilizing ability [21-23]. While many studies have largely focused on oxidative stress and ROS, the purpose of this paper is to address the impact of RNS on male infertility. A consolidation of the available information on RNS will allow the development of potential anti-oxidant treatments for conditions associated with nitrosative stress. Overall, through these aforementioned treatments and improvements in assisted reproductive techniques, favorable results for subfertile couples can be achieved [15,16].

### Sources of reactive nitrogen species

**Figure 1:**
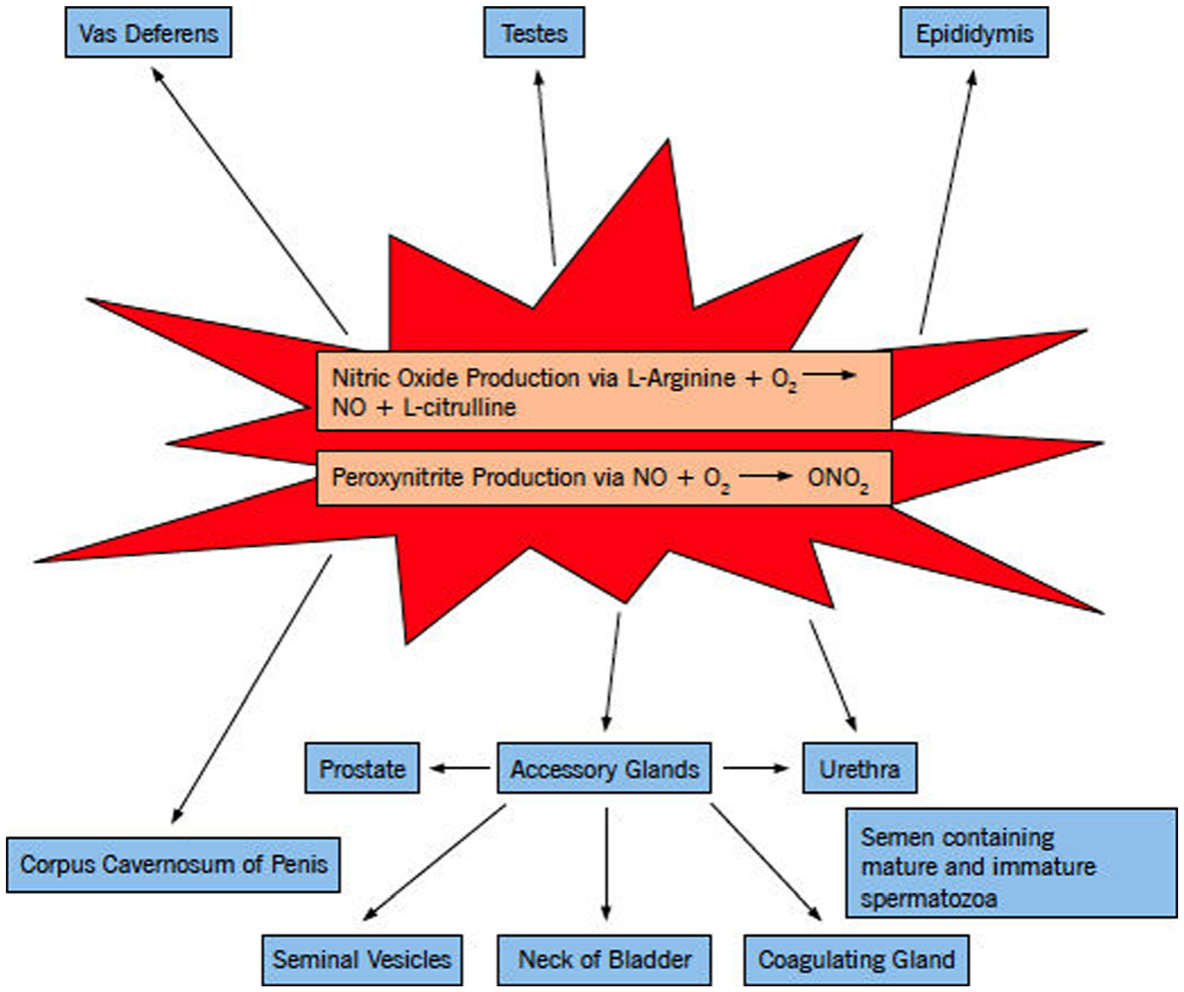
**Sources of reactive nitrogen species.** It illustrates the various locations of RNS throughout the male reproductive system. Specifically, these sources can be broken down by structure and various cell types as shown above.

Within the human body, a myriad of sources produce RNS, such as mesangial cells, smooth muscle cells, platelets, and hepatocytes [12,24,25]. However, RNS is especially prominent in different areas of the male reproductive system and these sources can be categorized by structure and various cell types such as seminal ejaculate, accessory glands, epididymis, penis, testes, and ducts [See Table 1; Figure 1. 

**Table 1:** **Location of nitric oxide**/**nitric oxide synthase in the male reproductive system**

**Source**	**Ref.**
**Seminal Ejaculate**	[21,22]
Mature and immature spermatozoa	
**Accessory glands**	[24-28]
Coagulating gland, urethra, penis, neck of bladder, prostate, seminal vesicles	
**Epididymis**	[27,29]
Caput, corpus, cauda	
**Penis**	[26,30]
Corpus cavernous	
Pelvic plexus	
Cavernous nerves and their terminal endings within the corporeal erectile tissue	
Branches of the dorsal penile nerves, nerve plexuses in the adventitia of the deep cavernous arteries	
**Testes**	[20,24,25,27,29,31]
Leydig Cells in adolescent males	
Round cells from different stages of spermatogenesis	
Infiltrating leukocytes, specifically neutrophils in conditions such as leukocytospermia	
Epithelial cells	
Endothelial cells	
Smooth muscle cells	
Macrophages in the intertubular area of the seminiferous tubules	
**Ducts**	[24-27,29]
1. Ejaculatory duct	
2. Vas Deferens	

### Mechanism of formation of nitric oxide

Nitric Oxide (NO) is produced from L-arginine via Nitric Oxide Synthase (NOS). It requires oxygen and a number of cofactors such as nicotinamide adenine dinucleotide phosphate (NADPH), flavin mononucleotide (FMN), flavin adenine dinucleotide (FAD), calmodulin, and calcium, resulting in the formation of NO as well as a byproduct known as L-citrulline [10-12,24,25]. There are three forms of NOS which exert their effect through protein-protein interactions and catalyze the aforementioned reaction: 1) endothelial NOS (eNOS), 2) inducible NOS (iNOS), and 3) neuronal NOS (nNOS). Each isoform has a reductase domain that contains a compound known as tetrahydrobiopterin (BH4), which is essential for efficient production of NO [24,25]. Interestingly, a testis-specific subclass of nNOS, known as TnNOS, has been recently identified as a major contributor to the formation of NO [31-34]. TnNOS is found to localize solely in the Leydig cells of the testis, thereby suggesting its involvement in steroidogenesis. With regard to eNOS and inducible NOS, both have been shown to be structurally associated with proteins such as occludin, actin, alpha-tubulin, vimentin, suggesting their importance in controlling tight junctions in the testis. Moreover, studies have illustrated that both eNOS and iNOS are involved in germ cell apoptosis. This is shown by the fact that eNOS has been linked to degenerating germ cell lines while iNOS has been associated with maintenance of germ cell number in the seminiferous epithelium. When considering iNOS alone, studies have shown that it partly mediates alpha-fodrin proteolysis, promoting germ cell necrosis.

Usually, iNOS is expressed in most cells only after induction by immunologic and inflammatory stimuli, such as peritoneal macrophages and macrophage products, including interleukin-1 and tumor necrosis factor-alpha [20,31,32]. However, it has been shown that this form of NOS can be induced by factors released from round spermatids, implicating a regulatory role of germ cells on Sertoli and Leydig cell NOS function [20,34]. Overall, these three isoforms are found in various cells of the testis, including Sertoli cells, germ cells in the seminiferous epithelium, Leydig cells, myofibroblasts, myoid cells, endothelial cells, and spermatozoa. The ubiquitous presence of eNOS, iNOS, and nNOS in the testis is indicative of the importance of NOS for spermatogenesis [9]. Aside from NOS, there are a variety of other compounds and biochemical reactions that produce NO in the body. Namely, studies have linked the generation of NO to the rate-limiting enzyme glucose-6-phosphate dehydrogenase as well as the NADPH-producing pentose phosphate pathway [35].

Furthermore, glucose has been shown to indirectly produce NO by stimulating the pentose phosphate pathway as well as the conversion of L-arginine to L-citrulline. Other studies have indicated that NO can regulate its own activity via a feedback inhibition mechanism [24,25]. While excessive levels of NO undoubtedly damage reproductive organs, it is nevertheless one of least potent of the RNS. In particular, the reaction of NO with the superoxide anion results in the formation of a more noxious oxidant, peroxynitrite [10-12,17,18,35]. Highly toxic to sperm, peroxynitrite and its breakdown product, Peroxynitrous acid (HONOO), are capable of inducing peroxidative damage and nitrosation of tyrosine molecules that facilitate signal transduction [24,25]. These cytotoxic effects often occur via the interaction of peroxynitrite and HONOO with nucleic acids, proteins, and lipids within the spermatozoa either through direct oxidative reactions or indirect, radical-mediated mechanisms [20,24,25]. Usually, the formation of peroxynitrite occurs only when NO has reached toxic levels and begins to compete with superoxide dismutase for the scavenging of superoxide [9]. Other important RNS include nitrosyl-containing compounds. Specifically, NO reacts with the thiol groups of proteins forming biologically active and stable S-nitrosyl compounds. Researchers report that production of the isoform, dinitrosyl iron cysteine, could be a possible mechanism for NO transport.

Additional pathways of nitric oxide transport can be generated via binding of NO to iron-sulfur clusters, formation of nitrotyrosines, and binding of NO to heme-containing proteins of the respiratory chain [20]. Overall, by developing a better understanding of the mechanisms and effects of RNS on the body, novel therapeutic strategies can be formulated to remove these toxic compounds and thereby alleviate their harmful effects [15].

### Role of reactive nitrogen species

#### Function of reactive nitrogen species in signal transduction

At physiologic levels, RNS are crucial for various functions within the male reproductive system. Although literature fails to elaborate on the exact levels of RNS that result in pathogenesis, studies have shown that concentrations below one micromolar play an important role in the regulation of a variety of signaling pathways [15,16,20,31,32]. At low levels, less than one micromolar, NO directly interacts with soluble guanyl cyclase to stimulate synthesis of cyclic guanosine monophosphate (cGMP), which in turn, activates cGMP regulated phosphodiesterase, protein kinase G, and cyclic nucleotide-gated channels. Additionally, minimal concentrations of nitric oxide, less than one micromolar, can induce mitogen activated protein (MAP) kinase signaling pathways. These pathways relay information to effectors, amplify signals, coordinate incoming information from other signaling pathways, and allow for a variety of response patterns [20].

### Role of reactive nitrogen species in the blood-testis-barrier

While NO is clearly important for intracellular signaling pathways, it is also involved in the regulation and assembly of tight junctions within the blood-testis barrier. This barrier creates an environment in which germ cells develop via changes in the chemical composition of the luminal fluid. The blood-testis barrier also prevents passage of toxic substances into the seminiferous tubules. Specifically, NO controls the timely opening and closing of this barrier, which has been found to be essential for the processes of spermatogenesis, germ cell maturation, and development [20,31,32]. Conversely, studies show that NOS inhibitors facilitate the assembly and tightening of the Sertoli cell-tight junction barrier, preventing the passage of spermatocytes and their complete maturation from spermatogonia to haploid spermatozoa across this barrier [20]. Hence, by understanding the importance of nitric oxide in mediating tight junction dynamics, appropriate male contraceptives can be targeted to inhibit the maturation of the spermatozoa across the blood-testis barrier, thereby preventing the formation of viable sperm for pregnancy [20,31,32].

### Function of reactive nitrogen species in the immune system and male reproductive organs

Other physiologic roles of RNS include mediation of cytotoxic and pathological events, production of hormones, and facilitation of inflammation via prevention of platelet aggregation and adherence of neutrophils to endothelial cells [10-12,24,25,36]. RNS also contributes to normal vascular tone, which is especially important during an erection via the NO/guanyl cyclase/cyclic GMP signaling pathway [4-6,17,18,26]. Moreover, studies have found that NOS innervates the sympathetic preganglionic neurons from the spinal cord to the penis, suggesting that NO may affect penile erection at several neuronal levels [24,25]. In the urogenital tract, NO allows for appropriate function of the penis, urethra, and bladder, thereby suggesting its role as a physiological mediator of peripheral autonomic activity [27]. Thus, it appears that physiological amounts of NO aid in many of the baseline functions of the male reproductive system.

### Positive effects of nitric oxide on sperm parameters

While physiological levels of nitric oxide are important in a variety of general body functions, they also are essential for conducting various sperm functions such as capacitation, acrosomal reaction, zona pellucida binding, as well as sperm motility, morphology, and viability [7-12]. Capacitation is an ongoing priming process that spermatozoa undergo in the female genital tract. It involves NO-mediated tyrosine phosphorylation of two sperm proteins and is marked by the influx of bicarbonate and calcium, efflux of cholesterol, and increase in pH and cyclic AMP [7-9,21,22]. Reports suggest that incubation of sperm with low concentrations of an NO-releasing compound, sodium nitroprusside, increases the percentage of capacitated sperm (p=0.0007) measured by the acrosome reaction [10-12,37]. Additionally, NO has been demonstrated to modulate lipoxygenase and cyclooxygenase activities during capacitation, implicating nitric oxide in sperm function [10-12].

**Figure 2:**
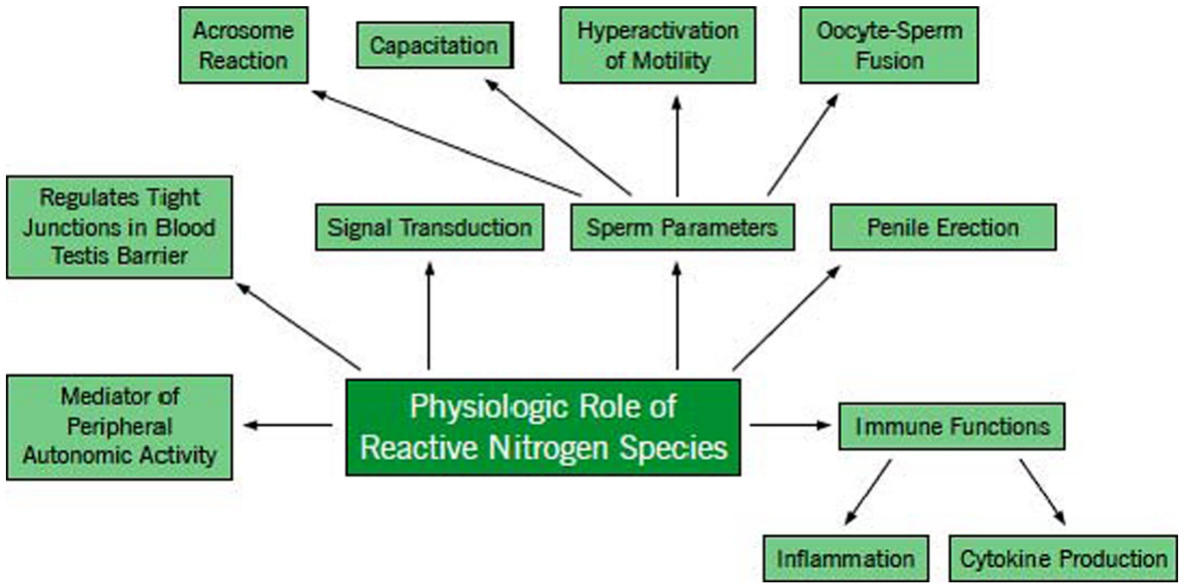
**Physiologic role of reactive nitrogen species.** It shows the essential role RNS plays in the human body. At physiologic levels, RNS helps to stimulate the immune system, maintain normal sperm parameters, and carry out general reproductive functions in the male body as shown above.

In order for capacitation to occur, spermatozoa must achieve two events for proper fertilization: hyperactivated motility and the acrosome reaction [10-12,37,38]. In particular, hyperactivation of sperm is characterized by high amplitude, asymmetric flagellar movement, non-linear motility, and a propulsive force to penetrate the cells of the oocyte [7-9,39]. While many experiments demonstrate that physiological levels of NO increase the motility of spermatozoa and excess amounts decrease motility, some studies have failed to show significant changes in sperm motility in the presence of this compound. Specifically, two studies showed that NO/NO variant concentrations <1 micromolar increased sperm motility, while six of studies showed a decrease in sperm motility at concentrations >1 micromolar. Additionally, one study showed that concentrations above or below one micromolar did not effect on sperm motility. Interestingly, one study in which NO concentration exceeded the physiologic parameter of one micromolar demonstrated increased motility, while one study in which NO concentrations fell below one micromolar decreased motility [See Table 2; Figure 2. 

**Table 2:** Effects of nitric oxide levels on sperm parameters

**Sperm parameter**	**Concentration of sperm or NO/NOS/NO inhibitor/releaser**	**Results**	**Ref.**
*Motility*	50-100 nM eNOS	1. Aberrant patterns of sperm eNOS expression associated with decreased sperm motility (r = −0.46; p<.05).	[26]
		2. At low concentrations, NO improved post- thaw sperm motility.	
*Motility*	10^-5^ M L-NAME	1. In the presence of NO inhibitor, L-NAME, the percentage progressive motility, average path velocity, straight linear velocity, and curvilinear velocity were significantly reduced after 30 min.	[40]
*Motility*	SNP levels in infertile males with leukocytospermia (6.58 ± 0.5.6 μM); infertile males without leukocytospermia (5.51 ± 0.25 μM) vs. Control (3.91 ± 0.16 μM)	1. There was a significant correlation between the NO_2_ concentration and sperm motility (r = 0.33; p<0.0005).	[41]
		2. SNP reduced the sperm motility in a dose- and time-dependent manner (p<0.0001).	
*Motility*	0-3 nmol x 10^6^ NO	1. Higher NO concentrations result in lower total percentage of sperm motility (p<0.0007).	[38]
*Motility*	> 20 x 10^6^ sperm/mL	1. Addition of SNP decreased mouse sperm motility, without any change in hyperactivation.	[42]
		2. The NOS inhibitor, L-NAME, and the NO scavenger, methylene blue inhibited sperm motility (p<.0.005).	
*Motility*	GSNO (100 nmol/L)	1. A 20 minute incubation of nitric oxide with NO releasing compounds (GSNO, PTIO, ODQ, 8-Br-cGMP, and Rp-8-Br-cGMPSs) did not alter the progressive motility of human sperm (p<0.05).	[39]
	PTIO (100μmol/L)		
	ODQ (50μmol/L)		
	8-Br-cGMP (1mmol/L)		
	Rp-8-Br-cGMPSs (10μmol/L)		
*Motility*	50 nM and 100 nM SNP	1. The maintenance of percent motility at 3 hours post-thaw was significantly improved in SNP treated samples (p<0.05)	[43]
*Motility*	5μM GSNO	1. The NO donor, GSNO, significantly increased progressive motility (77, 78, and 78% vs 66, 65, and 62% of the control)	[44]
		2. A similar effect was obtained with the NO donor sperm,NONOate, after 30 and 60 min.	
*Motility*	SNP (0.25-2.5 mM)	1. NO decreased sperm motility (r = 0.740; p<0.01).	[35]
	SNAP (0.012-.6 mM)		
*Motility*	SNAP 0-1.2 nmol/10^6^ spermatozoa	1. A positive correlation was seen between the concentrations of NO and the percentage of immotile spermatozoa (p<0.01).	[24,25]
*Motility*	10^-6^ to 10^-4^ M SNP	1. The percentage of motile sperm, progressive motility, and concentration of motile cells were all significantly reduced with all doses of SNP (p<0.005).	[45]
*Morphology*	Good morphology ≥ or equal to 14% normal sperm.	1. No significant difference was observed between NO production and sperm morphology (good or poor).	[39]
	Poor morphology <14% normal	2. No association was found between poor semen quality and elevated levels of basal NO production.	
*Morphology*	Good morphology ≥ or equal to 14% normal sperm	1. A positive correlation was shown between concentrations of seminal plasma NO and defects in sperm morphology (r = 0.4; p<0.05).	[46]
	Poor morphology <14% normal	2. Low levels of NO within the seminal plasma has been associated with defects in sperm morphology (r = 0.4; p<0.05).	
*Viability*	10^-6^-10^-4^ M SNP	1. Sperm viability in SNP treated sperm did not differ significantly from that of control sperm (p>0.05).	[45]
*Viability*	SNP (0.25-2.5 mM)	1. NO has been found to reduce sperm viability (p<0.05).	[35]
	S-nitroso-N-acetylpenicillamine (SNAP: 0.012-0.6 mM)		
*Viability*	10^-5^ M L-NAME	1. Sperm viability did not decrease in the presence of L-NAME, a nitric oxide synthase inhibitor.	[47]
*Viability*	50-100 nM NO	1. At low concentrations, NO improves post-thaw sperm viability.	[36]
*Viability*	>40 x 10^6^ sperm/mL	1. NO released by SNP has been shown to play a role in the maintenance of sperm viability after cryopreservation.	[38]
*Viability*	10^-4^M SNP	1. NO has been shown to decrease sperm viability	[45]

L-NAME = NG-nitro-L-arginine methyl ester.

SNAP = S-nitroso-N-acetylpenicillamine.

SNP = Sodium Nitroprusside.

GSNO = *S*-Nitrosoglutathione.

PTIO = 2-Phenyl-4,4,5,5-tetramethylimidazoline-3-oxide-1-oxyl.

ODQ = 1*H*-[1,2,4]Oxadiazolo[4,3-*a*]quinoxalin-1-one.

8-Br-cGMP = 8-Bromoguanosine- 3', 5'- cyclic monophosphate.

Rp-8-Br-cGMPSs = 8-Bromoguanosine- 3', 5'- cyclic monophosphorothioate, Rp-isomer.

Closely intertwined with motility, the acrosome reaction is also influenced by the amount NO in the body. Release of proteolytic enzymes from the acrosome or cap of the spermatozoa occurs as the sperm approaches the ovum. These enzymes create an opening in the glycoprotein layer, allowing the sperm to appropriately bind and fuse with the mature egg. Studies have shown that there is an increased percentage of sperm undergoing the acrosome reaction in the presence of the NO-releasing compound, sodium nitroprusside [10-12,37,42]. In addition to its significance in mediating the acrosomal reaction, physiologic levels of nitric oxide are also necessary for zona pellucida binding [38]. Specifically, incubation of spermatozoa with low levels of sodium nitroprusside (10^-7^ - 10^-8^ M) increased the binding of sperm plasma membrane to the membrane of the ovum [10-12]. Moreover, NO also contributes to normal sperm morphology, which has been shown to aid in the accurate prediction of fertility status and pregnancy outcomes during assisted reproductive techniques [48].

### Nitric oxide and its effects on genetic integrity

Since normal levels of NO play an integral role in carrying out these aforementioned functions, it follows that excess levels of NO can disrupt these signaling pathways and reactions. More specifically, concentrations greater than one micromolar have been associated with a variety of detrimental effects. For instance, the mitochondrial or nuclear DNA of spermatozoa can become severely damaged via the processes of deamination, oxidation, or nitration [20,31,49]. DNA damage is especially injurious given the variety of unfavorable fertility outcomes that can result from such harm. Specifically, impaired conception rates, increased incidence of abortion, and genetic defects in offspring have all been linked to various forms of damage in the spermatozoan genome. Furthermore, damage may include high frequency of double and single stranded breaks, inhibition of mitochondrial respiration and DNA synthesis, chromosomal rearrangements, chromatin cross-linking, modifications of bases, and chromosome microdeletion [15,16,21,22,35].

### Nitric oxide and its role in apoptosis

Other problems associated with pathological levels (10^-4^M) of NO include germ cell degeneration via apoptosis [20]. Usually, apoptosis is a normal and necessary response of the male reproduction system to eliminate abnormal spermatozoa. However, high levels of NO damage the mitochondrial membrane of the sperm, inducing the release of cytochrome-c and the activation of the caspase cascade to stimulate excess apoptosis [15,16].

### Effect of nitric oxide on steroid production

Studies have also shown that pathologic amounts of NO, greater than one micromolar, inhibit Leydig cell steroidogenesis. While the production of male steroids is important for development of secondary sexual characteristics and semen production, it can be hindered by the disruptive effect of excess NO on cytochrome P450, which is a cholesterol side-chain cleavage enzyme [20,24,25,50]. Eventually, the lack of steroids, such as testosterone and dihydroxytestosterone, can result in male infertility and a variety of diseases such as hypogonadism, type II diabetes, and metabolic syndrome [50].

### Excess nitric oxide and sperm lipid peroxidation

Toxic levels of NO, greater than one micromolar, have been implicated in lipid peroxidation of the polyunsaturated fatty acids (PUFA) within the sperm plasma membrane. Specifically, PUFA are very susceptible to the attack of RNS because their chemical structure contains hydrogen that can be easily abstracted. Abstraction by NO triggers a cascade of reactions, resulting in the production of a free radical that can be further oxidized to form more free radicals. This NO-mediated chain reaction is known as lipid peroxidation [15,16,51-53]. Therefore, functions of nitric oxide are highly dependent on the concentration of NO present in the male reproductive system.

### Negative effects of nitric oxide on sperm parameters

**Figure 3:**
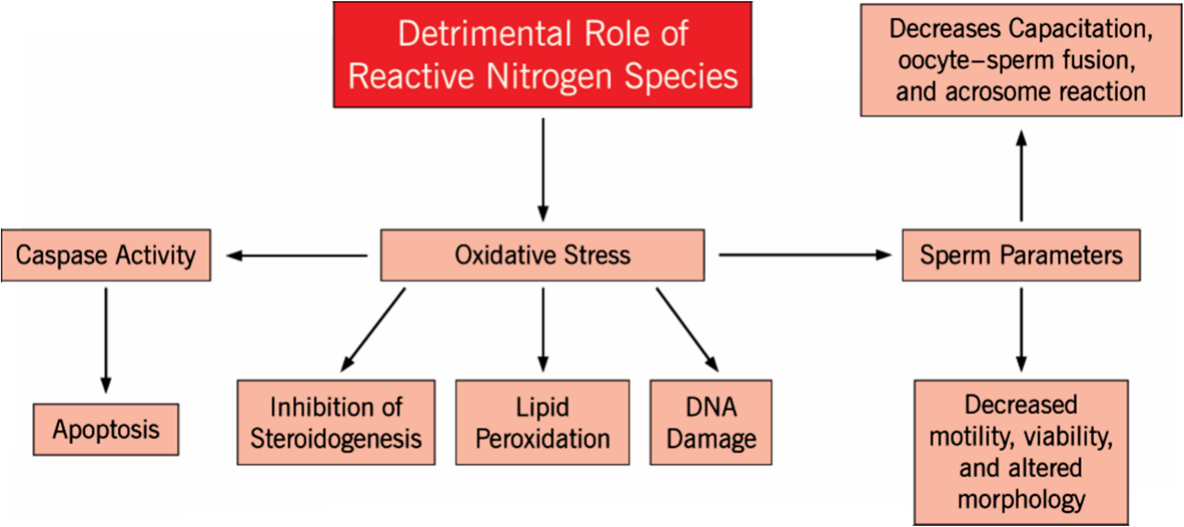
**Detrimental role of reactive nitrogen species.** This figure illustrates the harmful role RNS plays in the human body. At toxic levels, RNS can cause a variety of pathological effects on sperm parameters and normal cellular functions as shown above.

Although studies suggest that normal levels of NO help maintain the physiologic function of the male reproductive system, higher levels have been shown to be detrimental to the various sperm parameters mentioned in the previous paragraph. Specifically, researchers report that the number of sperm bound to the zona pellucida in the presence of 10^-4^M sodium nitroprusside was significantly less than the control group that was treated without NO. At this same concentration of sodium nitroprusside, a considerable decrease in the viability of sperm was seen; however, a few reports failed to show any significant effect of NO on this sperm parameter [45,54] Experiments have also indicated that toxic amounts of NO result in uncharacteristic sperm appearance or morphology [See Table 2[46]. However, similar to viability a few studies illustrated no significant effect of NO on sperm morphology [39,54]. Furthermore, higher concentrations of NO (5.74 ± 1.01 microM/L) in infertile men are more likely to result in the inhibition of capacitation in comparison to the seminal plasma NO concentrations (3.88 ± 0.53 microM/L) of normal, fertile men. Also, in these infertile men, higher levels of NO were linked to a decrease in sperm metabolism [55]. Overall, it is important that the body’s natural defense mechanisms properly regulate the levels of NO in order to prevent the detrimental effects of this compound on sperm functions [See Table 2; Figure 3. 

### Pathological effects of nitric oxide

#### Leukocytospermia

Toxic levels of NO have been often coupled with leukocytospermia, characterized by the generation of oxidative stress and an excess of white blood cells in the seminal ejaculate (1 × 10^6^ wbc/ml) [45,56,57]. Usually a contributory factor to subfertility, leukocytospermia interferes with sperm motility and causes agglutination of spermatozoa [45]. Additionally, it results in the production of cytotoxic cytokines and may be an indicator of an underlying infectious condition. Overall, leukocytospermia is often considered a major source of OS and may be the reason for higher than normal levels of nitric oxide in the body [45].

### Varicocele

Given that physiological nitric oxide governs many important functions in the body, it is unsurprising that excessive or insufficient amounts of this species can contribute to a variety of diseases and disorders. In particular, high levels of NO have been associated with varicocele, which is one of the most common causes of male infertility. Varicocele is a disease characterized by swelling and widening of the veins in the pampiniform plexus along the spermatic cord, thereby inhibiting blood flow in this area [33,58,59]. NO, specifically formed from iNOS has been implicated in the many symptoms associated in varicocele, including testicular hypoxia, Leydig and germ cell dysfunction due to small vessel occlusion, elevation in scrotal temperature, diminished gonadotropin secretion, and testicular dysfunction. Despite these proposed disruptions in varicocele, the exact mechanistic pathway by which NO functions in this pathophysiology is unclear [33,34,58-61].

### Erectile dysfunction

NO influences the mechanism of erectile dysfunction. Erectile Dysfunction is defined as the inability to achieve or maintain erections sufficient for satisfactory sexual intercourse [13,14,31]. Erection is mediated by the soluble guanyl cyclase/cGMP/cGMP pathway that is activated by NO. This pathway involves the phosphorylation of a variety of proteins which leads to the relaxation of smooth muscle and filling of blood within the sinusoidal spaces of the penis. However, in erectile dysfunction, there may not be enough NO to activate this pathway because it undergoes a competing reaction with oxyhemoglobin or superoxide anion to form toxic peroxynitrite [13,14,31].

### Diabetes mellitus

Pathological levels of NO have been considered a causal factor in diabetes mellitus. Specifically, NO has been linked to beta cell death in the pancreas, suggesting its role in the hyperinsulinemia associated with Type I diabetes. As a result of this insulin resistance, there are increased levels of insulin in the bloodstream, which has been proposed to play a role in inhibiting spermatogenesis and male fertility [62,63].

### Measurement of nitric oxide

Due to the very short half-life and reactivity of NO, measurement of this compound has proved very difficult. Nevertheless, a variety of direct and indirect methods for detection of NO have been employed in the laboratory setting. Namely, these methods are of great importance due to the many clinical implications involved with excess amounts of NO in the body.

### Direct methods of nitric oxide measurement

#### Iso-nitric oxide probe

The Iso-NO probe is a direct NO sensor, with a probe located at the end of stainless steel sleeve. This sleeve consists of a working and counter electrode combination that is covered by a polymeric membrane, which is permeable to gas [64]. In particular, this membrane separates the semen sample from the electrode and removes interference from other dissolved gases. The removal of such interference confers a high degree of selectivity to the probe. Specifically, the reason that concentrations from one nanomolar to twenty micromolars of NO can be measured across the gas-permeable membrane is a result of oxidation that it undergoes as it passes through the working electrode. The oxidation of nitric oxide produces an electrical redox current that is proportional to the amount of NO around the polymeric membrane [39].

### Flow cytometry

Flow cytometry is a method by which the intensity of fluorescence indicates the amount NO present in the sample. Specifically, NO production can be measured via changes in fluorescence which occurs after nitrosation of the NO indicator dye, diaminofluorescein-2 Diacetate (DAF-2DA). A counter stain specific to DAF-2DA is also used in order to make structures more visible [65].

### Indirect measurement of nitric oxide spectrophotometric measurement

The Griess test is one of the most sensitive methods for detecting nitrite or nitrous acid. Specifically, the presence of these NO-containing compounds can be assessed by reacting them with sulfanilic acid. The resulting product is a diazonium compound, which combines with alpha-naphthylamine to produce pink azo dye that is highly absorbent. In particular, the formation of this dye can be used to measure amounts of any substance that will yield nitrite in known proportions [66,67]. The manner in which the amount of nitrogenous compounds absorbed by the azo dye is measured is called spectophotometry. This method utilizes an instrument known as the spectrophotometer, which is a device for measuring light intensity as a function of wavelength as light passes through a solution. With regard to measurement of nitric oxide via spectophotometry, the NO concentration of the sperm sample is calculated according to Beer’s Law. Specifically, the amount of NO is assessed by the graph produced by the spectrophotometer which relates absorbance to wavelength.

### High performance capillary electrophoresis

High performance capillary electrophoresis separates nitrogen containing compounds based on their mass to charge ratio [68]. This method measures both nitrate and nitrite within the plasma and can be measured in a single analysis with minimal sample preparation. This method is sensitive to both basal and physiological changes in plasma nitrate and nitrite levels [68].

One of the limitations of these indirect methods is that they cannot determine the real time NO-concentration within a localized area at high spatial and temporal resolutions [66-69].

### Bio-imaging and electrochemical techniques

These new techniques can allow for continuous visualization and measurement of local NO concentrations at high resolutions. The bio-imaging method utilizes an NO-specific fluorescent dye that is capable of binding with NO and fluoresces in a manner dependent on NO concentrations. The fluorescent images are obtained using a confocal laser scanning microscope. In the electrochemical technique, a microaxial NO-electrode and reference electrode is used to measure the amount of NO oxidized at the surface of the electrode apparatus. In order to successfully measure NO, a combination of a variety of analytical methods are needed to investigate the complex mechanisms and physiological effects of NO [28,67].

### Assessment of reactive nitrogen species by chemiluminescence

The chemiluminescence assay utilizes an instrument known as a luminometer, in addition to a probe known as luminol [12,15,16]. Specifically, this probe is used in measurement of redox activities as well as ROS/RNS production within spermatozoa via the production of a light signal. This signal is formed from the interaction of these free radicals with the probes and it is eventually converted to an electrical signal by the luminometer [50]. Additionally, the luminol probe has the capacity to react with a variety of extracellular and intracellular ROS/RNS at a neutral pH, and is not specific to any particular type of free radical species [12,15,16,50]. The results of this assay are expressed as 10^6^ counted photons per minute per 20 × 10^6^ sperm or relative light units/s/10^6^ sperm [12,15,16].

## Conclusions

This article describes both physiological and pathological roles of this unique subset of ROS. Studies have shown that a balance between RNS and antioxidants is undoubtedly important for a variety of functions in the male reproductive system, such as cell signaling, tight junction regulation, and production of hormones, capacitation, acrosomal reaction, sperm motility, and zona pellucida binding [4,15,17,20,27,31]. However, an excess of RNS can adversely affect reproductive potential by causing testicular dysfunction, decreased gonadotropin secretion, and abnormal semen parameters [24,49,51]. Because such excesses have been demonstrated in males with fertility problems and routine semen analysis has not been able to accurately predict IVF outcomes, it is imperative that novel strategies be developed in order to both assess and treat OS. One promising approach that has been proposed is an antioxidant regimen to combat excess RNS levels. Presently, however, favorable results of antioxidant treatment have only been demonstrated in patients with high levels of DNA damage in sperm. Thus, the effectiveness of antioxidant treatment should be treated with brid led optimism until further research has been conducted. Another possible option that may be therapeutically important in correcting erectile dysfunction and infertility is the utilization of NO synthesis inhibitors and donors. Hence, the determination of an ROS threshold and development of various treatments in response to increased OS will not only be beneficial from a scientific standpoint, but will also have great clinical significance as well. Since excess levels of NO have been associated with decreased fertilization rates, impaired embryonic development, high levels of abortion and increased morbidity in the offspring, understanding the role of RNS can potentially stem the increasing rates of male infertility and decreasing sperm quality that has been reported in recent years.

## Abbreviations

cGMP: Cyclic Guanosine Monophosphate; DAF-2DA: Diaminofluorescein-2 Diacetate; eNOS: Endothelial Nitric Oxide Synthase; FAD: Flavin Adenine Dinucleotide; FMN: Flavin Monoucleotide; HONOO: Peroxynitrous acid; iNOS: Inducible Nitric Oxide Synthase; MAP: Mitogen Activated Protein; nNOS: Neuronal Nitric Oxide Synthase; NADPH: Nictoninamide Adenine Dinucleotide Phosphate; NO: Nitric Oxide; NOS: Nitric Oxide Synthase; OS: Oxidative Stress; PUFA: Polyunsaturated Fatty Acids; RNS: Reactive Nitrogen Species; ROS: Reactive Oxygen Species; BH4: Tetrahydrobiopterin.

## Competing interests

The authors declare that they have no competing interests.

## Authors’ contributions

SD participated in the original idea, carried out the literature (medline) search, compilation of the information, drafting and finalizing the paper. KK participated in compilation of the information and critical review of the paper. RKS conceived the study, participated in the study design compilation of the contents and critical review of the paper. AA provided substantial contribution ranging from study idea, design, critical review of the final paper. All authors read and approved the final manuscript.
